# Editorial: Herbal medicines in pain management, volume II

**DOI:** 10.3389/fphar.2024.1364073

**Published:** 2024-02-21

**Authors:** Domenico V. Delfino, Wen-Long Hu, Yu-Chiang Hung, Ming-Yen Tsai, Hung-Rong Yen

**Affiliations:** ^1^ Section of Pharmacology, Department of Medicine and Surgery, University of Perugia, Perugia, Italy; ^2^ Department of Chinese Medicine, Kaohsiung Chang Gung Memorial Hospital and Chang Gung University College of Medicine, Kaohsiung, Taiwan; ^3^ School of Nursing, Fooyin University College of Nursing, Kaohsiung, Taiwan; ^4^ School of Medicine, Kaohsiung Medical University College of Medicine, Kaohsiung, Taiwan; ^5^ School of Chinese Medicine, China Medical University College of Chinese Medicine, Taichung, Taiwan; ^6^ Integration of Chinese and Western Medicine, China Medical University Hospital, Taichung, Taiwan

**Keywords:** herbal medicines, ethnopharmacology, musculoskeletal pain, neuropathic pain, cancer pain, acute pain, chronic pain

## Background

Pain, when it is pathological and therefore especially chronic, represents a huge health problem all over the world. The numbers tell us that the prevalence of chronic pain is 33.5%, or 105 million people, with a cost of more than 100 billion dollars a year. We can classify pain into acute (high intensity and short duration) and chronic (low intensity and long duration). Examples of acute pain include pain as a result of an injury or acute post-surgical pain. Chronic pain can be further divided into nociceptive, neuropathic, visceral, and mixed pains. Neuropathic pain, in turn, can be divided into central and peripheral. Some examples make us understand the spread of the chronic pain phenomenon: Osteoarthritis is a nociceptive pain that affects about 16 million people; migraine and diabetic neuropathy, central and peripheral neuropathic pain, respectively, affect 31 million people and 3.2 million people, respectively; An example of visceral pain is that of pancreatitis, and finally, an extremely common mixed pain is that of the lower back, which affects 55 million people. A number of medications are used to treat chronic pain, and there are many new therapeutic strategies that are being developed. These include new formulations of old drugs, new indications of old drugs, and drugs with new mechanisms of action. A good alternative to allopathic medications are herbal or plant-derived medications. We have already published the first volume on herbal medicines useful for the treatment of pain (Hu et al., 2022), and now we present the second volume with four more articles that deal with the treatment of specific cases of pathological pain.

Many drugs are currently available for the treatment of pain, including several categories of pharmaceuticals (acetaminophen, nonsteroidal anti-inflammatory drugs, antidepressants, antiepileptics, local anesthetics, and opioids). However, despite this large battery of drugs, their use is limited by the fact that one in four patients does not respond to therapies and by the numerous adverse effects such as, for example, poor tolerability, side effects, concern for long-term safety, potential for abuse, and discomfort with use. In addition, the utilization of analgesic drugs will continue to increase due to several factors, including an aging population, an increased prevalence of chronic pain conditions, newly approved products, and better education of doctors and patients about the possibility of treating pain (Queremel Milani and Davis, 2023). All this requires the search for new drugs that can be effective in patients resistant to conventional drugs and that allow adverse effects to be reduced as much as possible. Herbal medicine is a frequently sought-after kind of complementary and alternative medicine (CAM). Herbal medicine is now used by around twenty million individuals in the United States, generating an annual revenue of over 1.5 billion dollars and seeing a growth rate of roughly 25% each year (Jahromi et al., 2021).

Neuropathic pain is certainly the one that poses the greatest therapeutic problems. Chronic neuropathic pain conditions including idiopathic trigeminal neuropathic pain, post-herpetic neuralgia, central post-stroke pain, and complex regional pain syndrome. The medications that are currently most frequently used in these conditions are anti-convulsant medications such as pregabalin and antidepressant medications such as fluoxetine. Other drugs have been approved, some of which are plant-derived. For example, St John’s Wort, Ginger, Turmeric, Omega-3 Fatty Acids, Capsaicin, Thunder God Vine, Butterbur, Feverfew, and Willow Bark. However, there is an urgent need for other drugs to resolve conditions resistant to current drugs (Fisher and Clarkson, 2024).

## Overview of the articles included in this Research Topic

Neuropathic pain is addressed as a side effect caused by the cancer drug vincristine, the drug of choice for Hodgkin’s lymphoma, lymphoblastic leukemia, and non-Hodgkin’s lymphoma. Vincristine causes dose-dependent neuropathic pain. In an experimental mouse model, Usman et al. found that 5,7-dimethoxycumarin improved vincristine-induced neuropathic pain by acting directly or indirectly on 5-HT3 receptors, since the effects were abolished by ondasentron but not by naloxone. Further clinical trials will be needed to establish whether this compound may be clinically beneficial.

Inflammatory pain also needs new and safer weapons. Sometimes, the pain becomes chronic if the inflammation is not promptly resolved. In addition, it can persist even after the damage that caused the inflammation has been cured (Ronchetti et al., 2017).

The other three articles deal with inflammatory pain. The herbal formulation called Xue-Fu-Zhu-Yu-Tang (XFZYT) is derived from traditional Chinese medicine. Kuo et al. conducted a population study and concluded that this formulation is successfully used for different types of pain, such as chest pain, headaches, myalgia and myositis, lumbago, and neuralgia, neuritis, and radiculitis.

Zurfluh et al., based on their anti-inflammatory and analgesic properties, used *Bryophyllum pinnatum* to treat dysmenorrhea. The authors inferred from a systematic review that this plant possessed analgesic and anti-inflammatory effects in thirty-three studies, and in sixteen studies, *B. pinnatum* resulted in myometrial relaxation. Subsequently, five patients were treated, and all reported a reduction in pain, and 4 out of 5 reduced their intake of other analgesics during menstruation. *B. pinnatum* was also well tolerated. There will be a need for larger clinical trials to confidently document the actions reported in the work.

Jo et al. conducted an analysis on *Piper longum* L. (PL), piperaceae, and osteoarthritis (OA). On the basis of this analysis, some therapeutic targets of PL in OA, such as F2R, F3, MMP1, MMP2, MMP9, and PTSG2, have been identified. While molecular docking results predicted a strong binding affinity for piperlongumins, piperlongumins, and piperines, compounds present in PL. These predictions were confirmed by *in vitro* experiments in which PL inhibited LPS-induced pro-inflammatory mediators such as F2R, F3, IL-1β, IL-6, IL17A, MMP-1, MMP-2, MMP-9, MMP-13, NOS2, PTSG2, PGE2, and TNF-β. In addition, in a rat model of OA, PL inhibited cartilage degradation.

In conclusion, medicinal herbs may represent an alternative or may have an adjuvant role in current therapy. However, in many cases, clinical trials are needed to validate the encouraging results obtained preclinically.
